# SIRT2 regulates nuclear envelope reassembly through ANKLE2 deacetylation

**DOI:** 10.1242/jcs.192633

**Published:** 2016-12-15

**Authors:** Tanja Kaufmann, Eva Kukolj, Andreas Brachner, Etienne Beltzung, Melania Bruno, Sebastian Kostrhon, Susanne Opravil, Otto Hudecz, Karl Mechtler, Graham Warren, Dea Slade

**Affiliations:** 1 Department of Biochemistry, Max F. Perutz Laboratories, University of Vienna, Dr Bohr-Gasse 9, Vienna 1030, Austria; 2 Department of Molecular Biotechnology, University of Applied Sciences FH Campus Wien, Helmut-Qualtinger-Gasse 2, 1030 Vienna, Austria; 3 Institute of Molecular Pathology, Dr Bohr-Gasse 7, Vienna 1030, Austria

**Keywords:** ANKLE2, SIRT2, Cell cycle, Nuclear envelope

## Abstract

Sirtuin 2 (SIRT2) is an NAD-dependent deacetylase known to regulate microtubule dynamics and cell cycle progression. SIRT2 has also been implicated in the pathology of cancer, neurodegenerative diseases and progeria. Here, we show that SIRT2 depletion or overexpression causes nuclear envelope reassembly defects. We link this phenotype to the recently identified regulator of nuclear envelope reassembly ANKLE2. ANKLE2 acetylation at K302 and phosphorylation at S662 are dynamically regulated throughout the cell cycle by SIRT2 and are essential for normal nuclear envelope reassembly. The function of SIRT2 therefore extends beyond the regulation of microtubules to include the regulation of nuclear envelope dynamics.

## Abstract (precis)

**Summary:** ANKLE2 is a new SIRT2 substrate. ANKLE2 deacetylation by SIRT2 is required for normal nuclear envelope reassembly. ANKLE2 and SIRT2 depletion yield aberrant nuclear morphology also found in Parkinson's disease, progeria and aging.

## INTRODUCTION

Sirtuins are NAD-dependent deacylases and mono(ADP-ribosyl) transferases involved in the regulation of gene silencing, genome stability, cellular metabolism, autophagy, apoptosis and lifespan (Bosch-Presegué and Vaquero, 2015; Choi and Mostoslavsky, 2014). By acting on both histone and non-histone substrates, sirtuins tune protein expression levels and protein activity in accordance with the cellular energy status (Houtkooper et al., 2012). Sirtuins catalyse the transfer of acetyl or acyl groups from lysine residues of substrate proteins onto the ADP-ribose moiety of NAD (Feldman et al., 2012). Of the seven members in mammals (SIRT1–SIRT7), all but SIRT4 can deacetylate lysine residues by releasing 2′-*O*-acetyl-ADP-ribose (*O*AADPr) and nicotinamide (Feldman et al., 2012). SIRT4 has a weak mono(ADP-ribosyl) transferase activity, SIRT5 exerts robust desuccinylase and demalonylase activity, whereas SIRT6 has mono(ADP-ribosyl) transferase and demyristoylase activity (Choi and Mostoslavsky, 2014; Feldman et al., 2012).

SIRT2 is a canonical sirtuin often referred to as a microtubule deacetylase. SIRT2 colocalizes with the microtubule network, shuttles between the cytoplasm and the nucleus (Inoue et al., 2007; North and Verdin, 2007a), transiently associates with chromatin in early prophase (Vaquero et al., 2006), and colocalizes with centrosomes and the mitotic spindle during metaphase (North and Verdin, 2007a). In mitotic cells, SIRT2 is stabilized by phosphorylation (Dryden et al., 2003; North and Verdin, 2007b). Its tubulin deacetylase activity is negatively regulated by Furry, which binds to microtubules and promotes microtubule acetylation in the mitotic spindle by inhibiting SIRT2 (Nagai et al., 2013). SIRT2 deficiency causes reduced proliferation, centrosome amplification and aneuploidy (Kim et al., 2011).

Tubulin, p53, FOXO1, H4, anaphase-promoting complex (APC) coactivators CDH1 and CDC20, and BubR1 (also known as BUB1B) represent the few characterized SIRT2 substrates, which reflect its dynamic cellular localization. α-tubulin was the first identified SIRT2 substrate (North et al., 2003), but this protein is also deacetylated by another deacetylase, HDAC6 (Hubbert et al., 2002). SIRT2 and HDAC6, however, seem to act on different subsets of acetylated tubulin, as trichostatin A (TSA) treatment, which inhibits HDAC6 but not SIRT2, results in a general increase in tubulin acetylation, whereas an NAD-depleting reagent (FK866) forms a patterned increase in tubulin acetylation around nuclei (Skoge et al., 2014). Furthermore, SIRT2 regulates gene expression by deacetylating the transcription factors, FOXO1 (Jing et al., 2007), the p65 subunit of NF-κB (Rothgiesser et al., 2010) and p53 (Jin et al., 2008). By deacetylating H4K16 at the G2/M transition, SIRT2 stimulates H4K20 monomethylation mediated by PR-Set7 (also known as KMT5A) (Serrano et al., 2013; Vaquero et al., 2006). Defective H4K20 methylation gives rise to delayed replication, DNA damage accumulation in S-phase cells and alterations in heterochromatin structure (Serrano et al., 2013). In mitotic cells, SIRT2 positively regulates the APC (Kim et al., 2011) and the mitotic checkpoint kinase BubR1 (North et al., 2014). SIRT2 deficiency decreases APC activity and stabilizes the APC ubiquitylation substrate Aurora A, which might explain the centrosome amplification observed in cells from SIRT2-knockout mice (Kim et al., 2011). SIRT2 deficiency reduces BubR1 levels, whereas SIRT2 overexpression stabilizes BubR1 and promotes lifespan extension (North et al., 2014).

In addition to its role in the regulation of normal cell cycle progression, SIRT2 is also important for the stress response. Specifically, SIRT2 is part of a mitotic checkpoint mechanism that regulates the stress response by arresting cells at the G2/M border upon treatment with hydrogen peroxide, hydroxyurea or microtubule poisons (Inoue et al., 2007; Serrano et al., 2013; Zhang et al., 2013). By deacetylating CDK9 and ATRIP, SIRT2 facilitates recovery from replication stress (Zhang et al., 2016, 2013).

SIRT2 is implicated in the pathology of cancer and neurodegenerative diseases. It acts as a tumour suppressor, as SIRT2-knockout mice develop mammary tumours or hepatocellular carcinoma (Kim et al., 2011) and loss of SIRT2 facilitates tumour formation in a model of skin squamous cell carcinoma (Serrano et al., 2013). SIRT2 is highly expressed in the brain and promotes neurodegeneration by downregulating neuronal motility (Harting and Knöll, 2010).

In order to gain further insight into SIRT2 physiological functions, we examined the effect of SIRT2 on mitotic progression. Surprisingly, we found that silencing or overexpressing SIRT2 causes nuclear envelope defects. To identify new SIRT2 substrates that might lead to this phenotype, we undertook a dual proteomics approach based on tandem affinity purification (TAP) and proximity biotinylation (BioID). The majority of the newly identified SIRT2 interactors were found to be associated with the cytoskeleton and the endoplasmic reticulum (ER) network. We demonstrated that one of the top-ranking ER interactors, ankyrin and LEM domain-containing protein 2 (ANKLE2), is deacetylated by SIRT2. ANKLE2 is a recently identified regulator of nuclear envelope reassembly (Asencio et al., 2012), the depletion of which results in deformed nuclei reminiscent of the SIRT2 phenotype. We link these phenotypes by showing that SIRT2 directly regulates ANKLE2 acetylation at residue K302 and indirectly affects ANKLE2 phosphorylation at S662 – a prerequisite for unperturbed nuclear envelope reassembly.

## RESULTS

### SIRT2 depletion and overexpression give rise to aberrant nuclear morphology

**Fig. 1.**
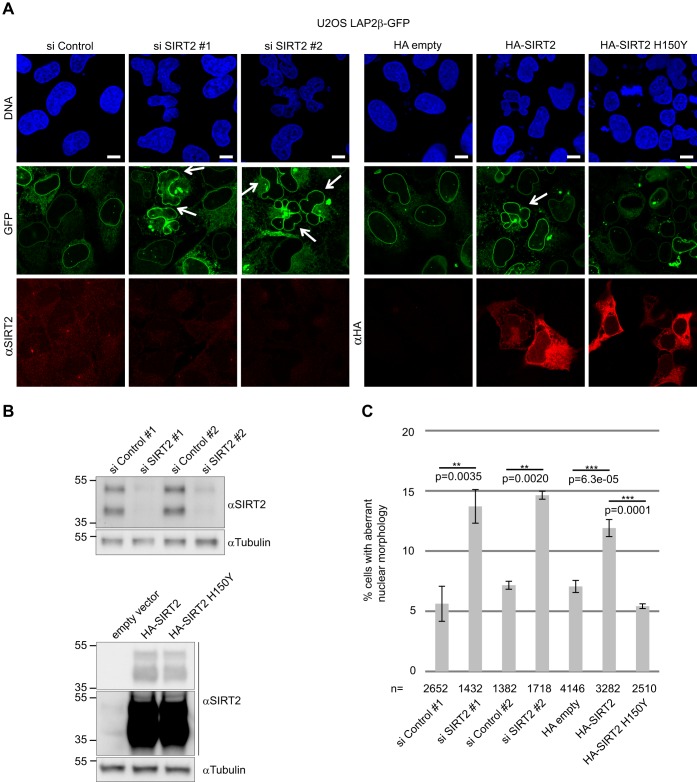
**Depletion or overexpression of SIRT2 causes nuclear envelope defects.** (A) U2OS cells stably expressing LAP2β–GFP were transfected with siRNA pools targeting SIRT2 (siSIRT2 #1, ON-TARGETplus; #2, siGENOME) or a non-silencing control (si Control), or with HA empty vector, or vectors expressing HA–SIRT2 or HA–SIRT2 H150Y. Arrows indicate cells exhibiting a lobulated nuclear envelope. Scale bars: 10 μm. (B) Western blots showing SIRT2 silencing efficiency and overexpression levels. Tubulin was used as a loading control. (C) Quantification of cells exhibiting a lobulated nuclear envelope 72 h after silencing SIRT2 or 48 h after overexpressing SIRT2. Mean±s.e.m. based on four independent experiments. **P<0.01; ***P<0.001 (two-tailed Student's *t*-test).

Silencing or overexpressing SIRT2 in a U2OS cell line stably expressing LAP2β as a nuclear envelope marker gave rise to nuclear envelope defects (Fig. 1A). The same phenotype was observed in U2OS cells stained for nuclear lamina proteins (lamin A/C) (Fig. S1A). The polylobed nuclear phenotype was observed in ∼14% of the cells 72 h after SIRT2 silencing and in ∼12% of the cells 48 h after SIRT2 overexpression (Fig. 1C; Fig. S1C). Two distinct short interfering RNA (siRNA) SIRT2 (siSIRT2) pools gave comparable phenotypes (Fig. 1). Overexpression of the SIRT2 catalytic mutant H150Y did not yield aberrant nuclei, which indicates that SIRT2 deacetylation activity is responsible for the polylobed phenotype (Fig. 1). These results suggest that any perturbation in acetylation homeostasis regulated by SIRT2 results in nuclear envelope shape defects.

### A combinatorial affinity purification and proximity biotinylation approach reveal new SIRT2 interactors

**Fig. 2.**
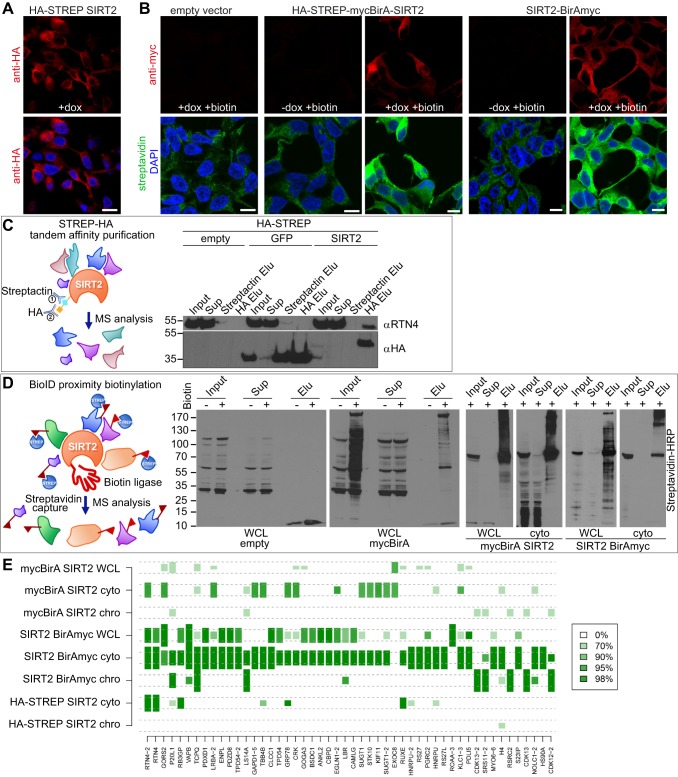
**Proteomics analysis of new SIRT2-binding partners identified by STREP-HA TAP or BioID.** (A,B) Immunofluorescence images of stable HEK293 cell lines expressing (A) HA-STREP–SIRT2 and (B) an empty vector control, HA-STREP–mycBirA–SIRT2 or SIRT2–BirAmyc under the control of a doxycycline-inducible promoter. Doxycycline (dox) was added for 24 h prior to processing for immunofluorescence microscopy using antibodies against the Myc tag or the HA tag (red), a fluorescently labelled streptavidin probe (green) and DAPI to counterstain DNA (blue). Negative controls were left uninduced; all media in (B) were supplemented with 50 µM biotin for 16 h to facilitate biotinylation. Scale bars: 10 µm. (C) A schematic presentation of STREP-HA TAP and western blot analysis of affinity purification of HA-STREP-tagged SIRT2. SIRT2 cytoplasmic fractions are first applied to a streptactin affinity column, bound proteins are eluted with biotin, and are then incubated with anti-HA magnetic beads and eluted with acidic glycine. 0.4% of the input, 0.4% of the supernatant (Sup), 2% of the streptactin eluate (Elu) and 50% of the anti-HA eluate were loaded on the gel. (D) A schematic presentation of BioID and streptavidin–HRP western blotting of BioID streptavidin pulldowns from whole-cell lysates (WCL) or cytoplasmic fractions (cyto) of N- and C-terminal SIRT2 BirA fusions. (E) A heat map of the top 50 SIRT2 interactors sorted by the descending global score. The hue indicates SAINT interaction probability. The height of a rectangle shows the proportion of replicates above the cut-offs (SAINT probability >0.98 and fold change >10). The global score is calculated based on the number of rectangles that go over the two dashed lines corresponding to a specific method. If the rectangle is over the two dashed lines, the corresponding method contributes one point to the global score.

To identify new SIRT2 interactors that might explain the nuclear envelope defects, we undertook a dual proteomics-based approach. STREP-HA tandem affinity purification (TAP) was used to reveal stable interactions (Glatter et al., 2009), whereas a proximity biotinylation (BioID) was used to detect weak or transient interactions (Roux et al., 2012). For the TAP approach, SIRT2 was tagged at the N-terminus with HA-STREP and stably integrated into Flp-In™ T-REX™ 293 cells under a doxycycline-inducible promoter (Fig. 2A). For the BioID approach, SIRT2 was fused to a promiscuous biotin ligase from *Escherichia coli* (BirA with the R118G mutation) at either the N- or the C-terminus, as it has been shown that the positioning of the BirA fusion yields different interactors (Van Itallie et al., 2013) (Fig. 2B). Both HA-STREP-tagged SIRT2 and mycBirA-fused SIRT2 were localized in the cytoplasm as previously reported for the endogenous SIRT2 (North et al., 2003; North and Verdin, 2007a), with particular enrichment on endomembranes (Fig. 2A,B; Fig. S2). Affinity immunoprecipitation of SIRT2 protein complexes using StrepTactin Sepharose (streptactin is a modified version of streptavidin) in the first step and anti-HA immunoprecipitation in the second step was performed as it reduces non-specific interactors and enables higher recovery of the tagged proteins compared to other TAP approaches (Glatter et al., 2009) (Fig. 2C). Cell lines stably expressing empty HA-STREP vector and HA-STREP–GFP were used as controls. Whereas TAP approaches require relatively high protein-complex stability for purification recovery, the BioID approach circumvents the issue of complex stability as proximal protein interactors are labelled covalently by biotinylation *in vivo* (Roux et al., 2012). Upon the addition of biotin to the cells, promiscuous BirA biotinylates the epsilon amine of exposed lysine residues on vicinal endogenous proteins within the range of 2–3 Å (Roux et al., 2012). Biotinylated proteins were selectively isolated by streptavidin pulldown and analysed by mass spectrometry (Fig. 2D). Cell lines stably expressing empty vector and mycBirA were used as controls. The empty vector control showed endogenously biotinylated proteins; freely diffusible mycBirA biotinylated primarily cytoplasmic proteins (Fig. 2D; Fig. S2C). Immunofluorescence microscopy showed cytoplasmic biotinylation of SIRT2-interacting proteins (Fig. 2B), whereas western blotting revealed numerous biotinylated proteins of primarily high molecular mass (Fig. 2D; Fig. S2D,E). Notably, N- and C-terminal BirA–SIRT2 fusions yielded distinct biotinylation patterns (Fig. 2D).

Our label-free mass spectrometry analysis was conducted on eight different samples: whole-cell lysates, cytoplasmic and chromatin fractions of N- and C-terminal BirA–SIRT2 fusions, and cytoplasmic and chromatin fractions of HA-STREP-tagged SIRT2. In order to discriminate high-probability interactors, we performed bioinformatics analysis of interaction probability based on SAINT-MS1 algorithms (Choi et al., 2012a) and fold change. As both the BioID and affinity purification approaches have inherent limitations and yield a certain number of false-positive hits, we generated a global score assignment strategy that rated the interactors based on stringent cut-offs: a SAINT probability of >0.98 and fold change >10 (see Materials and Methods for explanation of the global score). The global score strategy increases confidence that the highest scoring interactions are valid *in vivo*. Fig. 2E shows the top 50 proteins with the highest global score and their enrichment in eight different samples. SIRT2 interactors are primarily involved in the regulation of cytoskeleton and ER structure and dynamics; tubulin (β-4B chain; TBB4B), as a known SIRT2 interactor (North et al., 2003), ranked 15th in the global score (Fig. 2E).

As expected, BioID revealed a greater number of SIRT2 interactors compared to the TAP approach (Table S1). The C-terminal SIRT2–BirA fusion yielded a particularly abundant set of identified interactors, which suggests that the C-terminus is the primary SIRT2 interaction interface in the cytoplasm. Cellular fractionation also enhanced detection of SIRT2 interactors by reducing competition between specific and non-specific interactors for binding to the beads used for the pulldowns. Chromatin and nuclear interactors constituted only a minor portion of the BioID C-fusion interactome, but surprisingly represented the majority of the BioID N-fusion interactome, indicating that the N-terminus of SIRT2 is primarily involved in chromatin and nuclear interactions, such as those involved in transcription regulation and RNA processing. Although chromatin structure components and regulators are not strongly represented in the SIRT2 interactome, H4, as a published SIRT2 interactor (Vaquero et al., 2006), ranked 44th in the global score (Fig. 2E), whereas ATRIP (Zhang et al., 2016) was assigned a global score of zero as it was only identified in the chromatin fraction of the N-terminal BirA–SIRT2 fusion (SAINT score=0.846; fold change=6.38). Proteins involved in membrane trafficking were dominant in the TAP cytoplasmic sample. The overlap between the BioID and the TAP cytoplasmic interactomes was generally rather low (∼12%), which has also been previously reported for histone proteins and mediator complex subunits (Lambert et al., 2015). The low overlap between the two approaches might stem from their inherent limitations. Whereas the TAP approach traps only stable interactions, which are not expected for transient enzymatic reactions, the BioID approach is restricted by the inaccessibility of lysine residues on the surface of the interacting protein and the extended biotin labelling time, which might lead to the loss of biotinylated interacting partners due to protein turnover. We confirmed a subset of the newly identified SIRT2 interactors by co-immunoprecipitation from HEK293 cells: RTN4, coatomer complex proteins COPB1 and COPG2, the Rho-family GTPase RAC1, the NuRD chromatin remodelling complex proteins HDAC1 and MTA2, the chromatin condensation factors RCC1 and the condensin subunit CND2 (also known as NCAPH), as well as the kinetochore-associated protein SKAP (also known as KNSTRN), which is required for faithful chromosome segregation (Fig. S2F–H).

### SIRT2 deacetylates ANKLE2

**Fig. 3.**
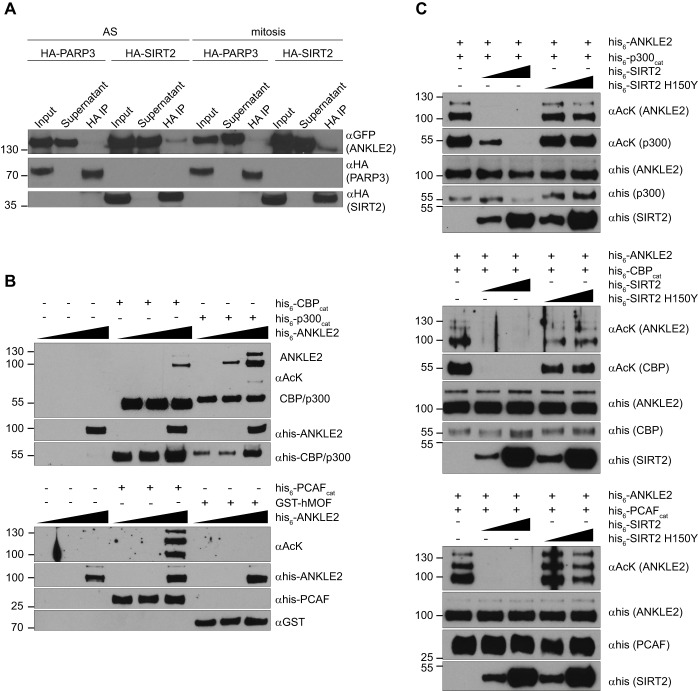
**SIRT2 deacetylates the regulator of nuclear envelope reassembly ANKLE2.** (A) Western blots of anti-HA co-immunoprecipitation (IP) from asynchronous and mitotic HEK293T cells overexpressing HA–SIRT2 and ANKLE2–GFP. HA–PARP3 was used as a negative control. (B) *In vitro* acetylation of recombinant ANKLE2 with 0.15 μg of p300, PCAF and CBP acetyltransferases. hMOF did not acetylate ANKLE2. 0.015, 0.15 or 1.5 μg of ANKLE2 was present in the loaded sample. (C) *In vitro* deacetylation of ANKLE2 by SIRT2. 1.5 μg of ANKLE2, 0.15 μg of acetyltransferase and 0.15/1.5 μg of SIRT2 or SIRT2 H150Y were present in the loaded samples.

ANKLE2, a recently identified regulator of nuclear envelope reassembly, ranked 22nd in the global score. We validated ANKLE2 as a SIRT2 interactor using HA–SIRT2 (Fig. 3A) or ANKLE2–GFP as bait (Table S2). Mass spectrometry analysis of ANKLE2–GFP interactors was performed in order to verify reciprocal interaction with SIRT2 in a semi-overexpression system where only ANKLE2 was overexpressed (Table S2). This analysis additionally revealed previously reported or new ANKLE2 interactors (see below). The ANKLE2–SIRT2 interaction was specific, as a control cytoplasmic protein, PARP3, did not interact with SIRT2 under the same co-immunoprecipitation conditions (Fig. 3A). Moreover, both ANKLE2 and SIRT2 localized within the ER (Fig. S3A,B). In order to determine whether SIRT2 directly interacts with ANKLE2 and deacetylates it, we performed *in vitro* acetylation and deacetylation assays (Fig. 3B,C). ANKLE2 was acetylated by the p300 (also known as EP300), CBP (also known as CREBBP) and PCAF (also known as KAT2B) acetyltransferases, but not hMOF (also known as KAT8) (Fig. 3B). Wild-type SIRT2, but not the catalytically inactive H150Y mutant, efficiently deacetylated all acetylated forms of ANKLE2 (Fig. 3C). SIRT2 also deacetylated p300 and CBP (Fig. 3C), as previously reported (Black et al., 2008). ANKLE2 (de)acetylation *in vitro* was analysed by mass spectrometry (Table S3). 35 out of 55 ANKLE2 lysine residues were acetylated *in vitro*, of which 31 sites were also deacetylated by SIRT2 (Table S3). P300 acetylated 31 sites, followed by CBP (21) and PCAF (19). This confirmed that ANKLE2 is a direct SIRT2 deacetylase substrate.

### SIRT2 modulates ANKLE2 acetylation and phosphorylation during the cell cycle

**Fig. 4.**
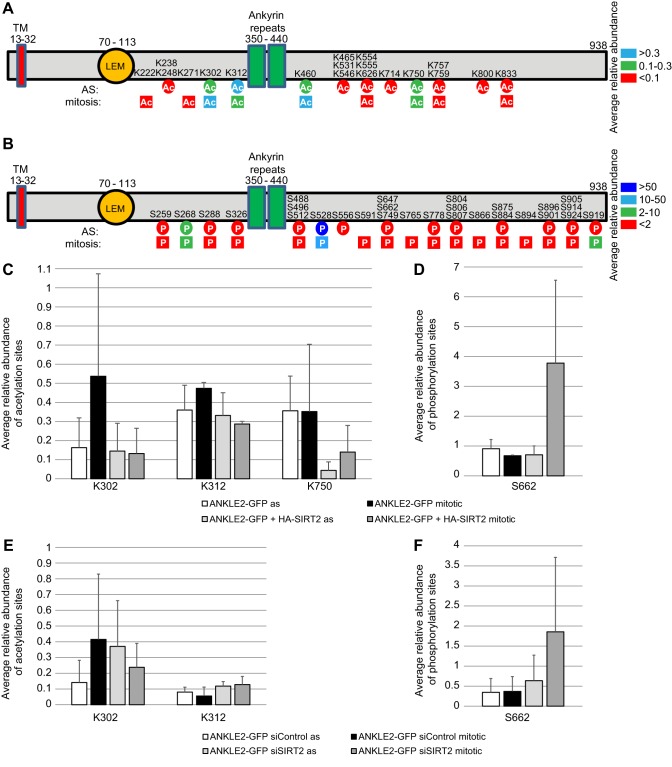
**SIRT2 regulates ANKLE2 acetylation and phosphorylation dynamics *in vivo*.** (A,B) ANKLE2–GFP (A) acetylation and (B) phosphorylation sites identified in asynchronous (circles) and mitotic (square) cells in three independent experiments. Acetylated lysine and phosphorylated serine residues are coloured according to their average abundance relative to unmodified peptides. (C–F) Changes in the relative abundance of (C,E) ANKLE2 acetylation sites and (D,F) a phosphorylation site upon SIRT2 overexpression (C,D) or silencing (E,F). Mean±s.e.m. values from two to four independent experiments are shown. The acetylation sites shown are characterized by a >0.1 relative abundance and a reproducible reduction in acetylation upon SIRT2 overexpression or increase in acetylation upon SIRT2 silencing. The phosphorylation site fulfils the criteria of a >1 relative abundance and a reproducible increase in mitotic phosphorylation. Relative abundance ratios show considerable variability due to inherent limitations of the shotgun mass spectrometry analysis of substoichiometric modifications.

Acetylation of ANKLE2 was also analysed *in vivo* in asynchronous and mitotically enriched HEK293T cells overexpressing ANKLE2–GFP (Table S4). ANKLE2 was acetylated at 19 out of 55 lysine residues (Fig. 4A), 16 of which were also identified *in vitro* (Table S3; K222, K460 and K465 were not found *in vitro*). Two out of the four most abundant acetylation sites – K302 and K312 – exhibited dynamic acetylation levels when comparing asynchronous to mitotic cell populations (Fig. 4C). Moreover, SIRT2 overexpression reproducibly reduced K302, K312 and K750 acetylation in mitotic and/or asynchronous cells (Fig. 4C). SIRT2 silencing led to reproducibly increased K302 acetylation levels in the asynchronous population, and increased K312 acetylation in both asynchronous and mitotic cells, whereas K750 acetylation was increased in only one out of four experiments (Fig. 4E). This indicates that SIRT2 might regulate ANKLE2 through deacetylation of these lysine residues.

We additionally identified 28 ANKLE2 serine phosphorylation sites that were of considerably higher relative abundance compared to acetylation (Fig. 4B). The most abundant phosphorylation site, S528, showed cell–cycle-regulated phosphorylation, being reduced in mitotic cells (Fig. 4B). Interestingly, S662 was the only site with consistently increased phosphorylation levels upon SIRT2 overexpression in mitotic cells (Fig. 4D). Surprisingly, SIRT2 silencing had the same effect on S662 as SIRT2 overexpression (Fig. 4F). These results indicate that SIRT2 directly regulates ANKLE2 by deacetylating K302, K312 and/or K750, and indirectly by regulating phosphorylation of S662.

### ANKLE2 depletion gives rise to deformed nuclei akin to those seen upon SIRT2 depletion

ANKLE2 was previously shown to regulate nuclear envelope reassembly in *C. elegans* by promoting dephosphorylation of the barrier-to-autointegration factor (BAF; also known as BANF1 in mammals) (Asencio et al., 2012). BAF is known as an essential DNA-binding protein, which interacts with a distinct structural motif termed the LAP2–Emerin–MAN1 (LEM) domain, thereby establishing connections between chromatin and a family of proteins containing a LEM domain (Jamin and Wiebe, 2015). Although the nuclear envelope breaks down in prophase, the ER network remains intact and recycles tubular material for nuclear envelope reassembly in telophase (Anderson and Hetzer, 2008). Nuclear envelope breakdown is coupled with chromatin condensation and phosphorylation of BAF, which causes it to dissociate from chromatin thereby promoting the release of nuclear-envelope-tethered chromatin from inner nuclear membrane proteins (Güttinger et al., 2009). By contrast, nuclear envelope reassembly occurs through recruitment of ER tubules to chromatin, RanGTP hydrolysis, nuclear envelope tethering to chromatin by inner nuclear membrane proteins, and restoration of BAF association with chromatin, which is mediated by dephosphorylation promoted by ANKLE2 (Asencio et al., 2012; Güttinger et al., 2009). Our mass spectrometry data confirmed BAF as an ANKLE2 interactor, corroborating that ANKLE2 is a bona fide LEM domain protein (Table S2).

**Fig. 5.**
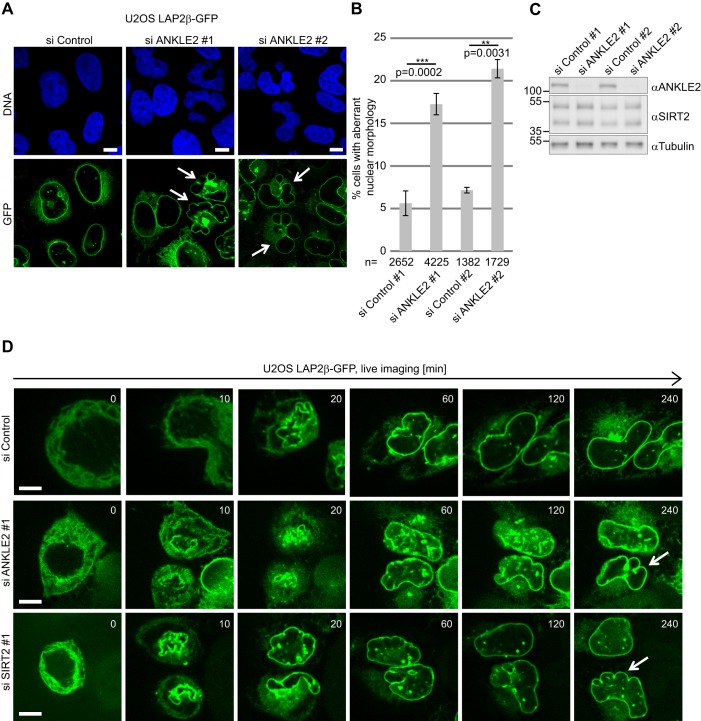
**ANKLE2 depletion results in defective nuclear envelope reassembly.** (A) U2OS cells stably expressing LAP2β–GFP were transfected with siANKLE2 (#1, ON-TARGETplus; #2, siGENOME) or a non-silencing control (si Control). (B) Quantification (mean±.s.e.m.) of cells exhibiting a lobulated nuclear envelope 72 h after silencing ANKLE2, based on four independent experiments. **P<0.01; ***P<0.001 (two-tailed Student's *t*-test). (C) Western blot showing siANKLE2 efficiency; tubulin served as a loading control. (D) Still images of live-cell imaging movies of U2OS LAP2β–GFP-expressing cells 48–72 h after transfection of siANKLE2 or siSIRT2. Arrows indicate cells with lobulated nuclear envelopes. Scale bars: 10 μm.

Silencing ANKLE2 (with siRNA; siANKLE2) yielded nuclear envelope defects as previously published (Asencio et al., 2012) (Fig. 5A). The polylobed nuclear phenotype was observed in ∼17% (siANKLE2 #1) or 21% (siANKLE2 #2) of U2OS LAP2β–GFP cells that were fixed and analysed 72 h after ANKLE2 silencing and resembled the SIRT2 phenotype (Fig. 5A,B, compare with Fig. 1). Live imaging of mitotic progression revealed that the polylobed phenotype was a result of impaired nuclear envelope reassembly in postmitotic ANKLE2- and SIRT2-depleted cells (Fig. 5D). Cells bearing polylobed shapes were unable to undergo a second round of mitotic division; the measured occurrence of the polylobed phenotype is therefore merely an underestimate as the dead cells are unaccounted for. Unlike for SIRT2, ANKLE2 overexpression had no effect on nuclear envelope reassembly (Fig. S3C,D). SIRT2 silencing or overexpression in siANKLE2-silenced cells did not rescue the polylobed phenotype (Fig. S4A,B).

### SIRT2 directly regulates ANKLE2-mediated nuclear envelope reassembly

**Fig. 6.**
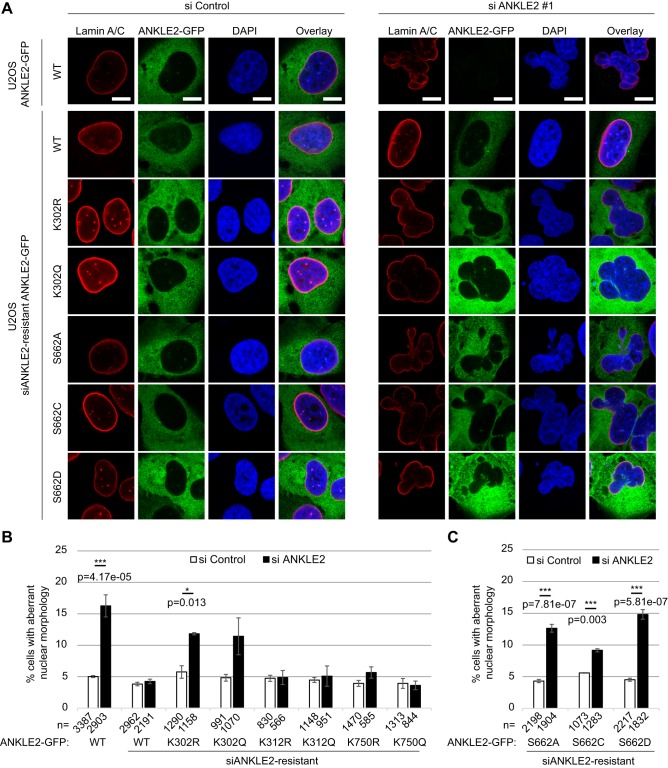
**SIRT2 directly regulates nuclear envelope reassembly by deacetylating ANKLE2.** (A) Immunofluorescence images of U2OS cells stably expressing ANKLE2–GFP or siANKLE2-resistant wild-type (WT) or acetylation or phosphorylation site mutant ANKLE2–GFP. The cells were transfected with siRNA targeting ANKLE2 or a non-silencing control (si Control) for 48 h, and co-stained for lamin A and C. ANKLE2 K302R and K302Q and ANKLE2 S662A, S662C and S662D mutants cannot rescue the polylobed phenotype caused by ANKLE2 depletion. Scale bars: 10 µm. (B,C) Quantification (mean±.s.e.m.) of the polylobed phenotype for ANKLE2 (B) acetylation and (C) phosphorylation mutants, based on two to four independent experiments. *P<0.05; ***P<0.001 (two-tailed Student's *t*-test).

Is the defective nuclear morphology in SIRT2-depleted cells directly caused by the disruption of the SIRT2–ANKLE2 complex? To address this question we tested whether mutations of the acetylation and phosphorylation sites that were found to be regulated by SIRT2 (Fig. 4) could rescue the polylobed phenotype caused by ANKLE2 depletion (Fig. 6). We generated U2OS cell lines with stable expression of ANKLE2–GFP and siANKLE2-resistant wild-type and mutant ANKLE2–GFP. The polylobed phenotype caused by ANKLE2 silencing was reproduced in U2OS ANKLE2–GFP cells (16.3±1.8%, mean±s.e.m.; Fig. 6B) compared to U2OS LAP2β–GFP cells (17.3±2.6%; Fig. 5B). K302, K312 and K750 were mutated to arginine (R) or glutamine (Q) to mimic the hypo- and hyperacetylated state, respectively. Whereas the siANKLE2-resistant ANKLE2–GFP wild-type, K312 and K750 mutants were able to fully rescue the polylobed phenotype caused by ANKLE2 depletion, K302R and K302Q did not rescue the phenotype (Fig. 6A,B), which indicates that the ANKLE2 K302 acetylation state is important for proper nuclear envelope reassembly following mitosis. However, the lower prevalence of the polylobed phenotype in K302R cells (11.8±0.2%) suggests that other ANKLE2 residues regulated by SIRT2 are additionally important for nuclear envelope reassembly. Indeed, perturbation of the phosphorylation dynamics of S662, which was also shown to be regulated by SIRT2 (Fig. 4D,F), through use of the hyperphosphorylation mutation (S662D), failed to rescue the polylobed phenotype (14.8±0.8%), whereas hypophosphorylation mutations only partially rescued the phenotype (12.6±0.6% for S662A and 9.2±0.2% for S662C) (Fig. 6C).

In addition, we tested whether SIRT2-mediated regulation of tubulin acetylation affected nuclear envelope reassembly directly (Fig. S4C,D). Although SIRT2 silencing doubled the number of cells with disorganized acetylated tubulin compared to the number seen in control cells (60% versus 31%), the proportion of polylobed cells with disorganized acetylated tubulin remained the same. Furthermore, although silencing ANKLE2 resulted in a more pronounced polylobed phenotype compared to silencing SIRT2, the proportion of cells with disorganized tubulin was lower (47%). We therefore conclude that microtubule disorganization is not the cause of the polylobed phenotype observed in SIRT2-depleted cells.

## DISCUSSION

Sirtuin deacylases enzymatically regulate a plethora of cellular functions depending on their cellular localization. SIRT2 shuttles between the cytoplasm and nucleus and regulates cell cycle progression. SIRT2-depleted cells exhibit reduced proliferation and centrosome amplification, which has been linked with the SIRT2-mediated regulation of the APC (Kim et al., 2011). In this study, we revealed a new function of SIRT2. We report that SIRT2 regulates nuclear envelope reassembly after mitosis through a newly identified SIRT2 interactor, ANKLE2.

**Fig. 7.**
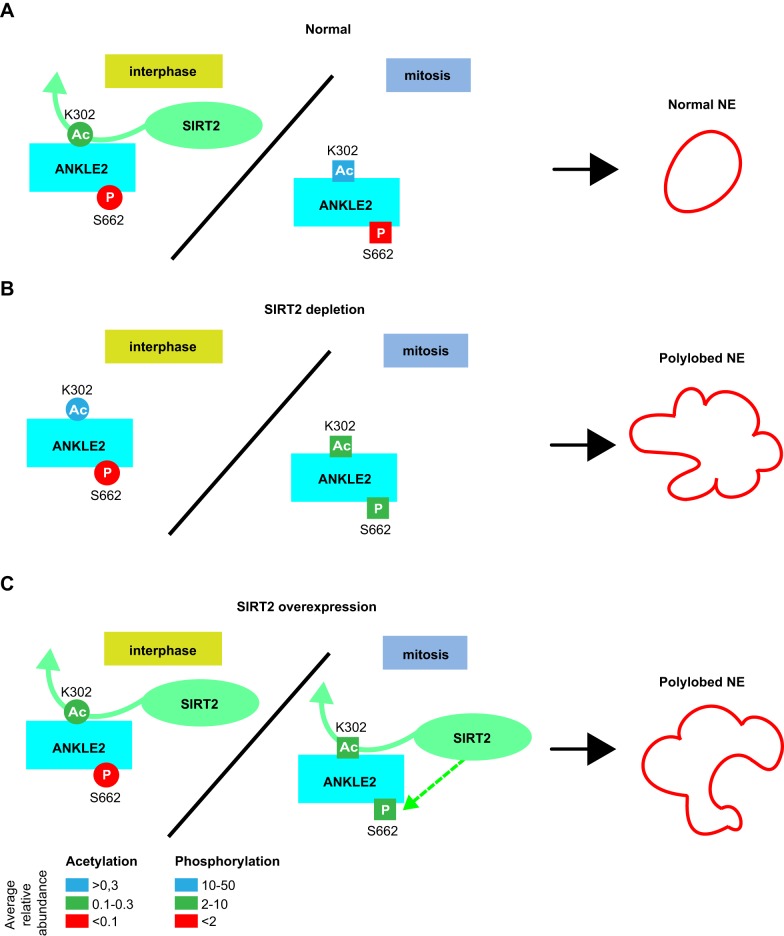
**A model of SIRT2-mediated cell cycle regulation of ANKLE2 acetylation and phosphorylation.** (A) Under normal conditions ANKLE2 K302 acetylation is low in interphase and high in mitosis, whereas S662 phosphorylation is low throughout the cell cycle. (B) SIRT2 silencing increases interphase but reduces mitotic K302 acetylation, and increases mitotic S662 phosphorylation. (C) SIRT2 overexpression reduces mitotic K302 acetylation and increases mitotic S662 phosphorylation. (B,C) Perturbation of ANKLE2 K302 acetylation levels and S662 phosphorylation upon SIRT2 depletion or overexpression result in a polylobed nuclear envelope phenotype.

As an ER-resident protein, ANKLE2 regulates the dephosphorylation of BAF by PP2A phosphatase at mitotic exit, which facilitates reassociation of BAF with chromatin and nuclear envelope reassembly (Asencio et al., 2012). We found that SIRT2 depletion or overexpression phenocopies the nuclear envelope reassembly defects caused by ANKLE2 depletion. Importantly, we identified an ANKLE2 acetylation site, K302, as being dynamically regulated by SIRT2. The acetylation state of ANKLE2 K302 was tightly regulated during the cell cycle, being low in interphase and high in mitosis. SIRT2 depletion by use of siRNA increased K302 acetylation in interphase, whereas SIRT2 overexpression decreased mitotic K302 acetylation. ANKLE2 K302R and K302Q mutants did not complement the polylobed phenotype caused by ANKLE2 depletion. In addition, we found that SIRT2 indirectly regulated ANKLE2 phosphorylation at S662 (by regulating an unknown kinase or phosphatase), which was also crucial for ensuring proper nuclear envelope reassembly (Fig. 7).

Our results indicate that SIRT2 deacetylates ANKLE2 K302 in interphase, whereas this specific SIRT2 activity is suppressed in mitosis (Fig. 7). SIRT2 is known to be regulated during mitosis through phosphorylation or Furry (see Introduction), which might affect its substrate-specific activity. When overexpressed, negative regulation of SIRT2 activity in mitosis might no longer be efficient, resulting in reduced mitotic ANKLE2 acetylation. Mitotic K302 acetylation was also reduced in SIRT2-silenced cells, perhaps due to induction of another deacetylase upon SIRT2 silencing. For example, HDAC1 and HDAC2 deacetylases form a complex, whereby silencing or knockout of one HDAC induces the activity of the other (Moser et al., 2014). The same might be true for SIRT2 and SIRT1 or another deacetylase.

Lack of complementation of the polylobed phenotype was observed with both types of acetylation mimetics (K302R and K302Q; K302Q was, however, not statistically significant). Acetylation mimetics have previously been reported to perturb protein–protein interactions or protein catalytic activity due to loss of electrostatic interactions or conformational changes induced by amino acid mimetics that are sterically distinct from lysine or serine. By way of example, SMC3 K105/106 RR and QQ acetylation mimetics have a similar effect on sororin binding (Nishiyama et al., 2010), whereas PGK1 K220R and K220Q both reduce its catalytic activity (Wang et al., 2015). Alternatively, if the protein function is regulated by distinct acetylation states depending on the cell cycle stage, as in the case of K302 acetylation levels being low in interphase and high in mitosis, any perturbation of this homeostasis through either the ablative or mimetic mutations would prevent complementation of the protein function.

Why do SIRT2 silencing and overexpression give rise to the same mitotic phenotype? On the one hand, mitotic ANKLE2 acetylated at K302 (K302ac) might be the only state important for ANKLE2 function in mitosis and K302Q might not act as a good acetylation mimetic (as was previously shown for the other proteins mentioned above). Hence decreased K302ac in mitosis, either when SIRT2 is overexpressed (direct effect) or depleted (indirect effect through another deacetylase), results in the polylobed phenotype. On the other hand, both low interphase and high mitotic ANKLE2 K302 acetylation states might be important for ANKLE2 function in nuclear envelope reassembly. High interphase K302ac due to SIRT2 depletion or low mitotic K302ac due to SIRT2 overexpression both result in the polylobed phenotype. If both high and low acetylation levels are needed this would explain why neither K302R nor K302Q, assuming that they are functional mimetics, can rescue the phenotype. Unlike SIRT2, ANKLE2 overexpression did not yield the same phenotype as its depletion. In conclusion, both loss and excess of SIRT2 activity perturb nuclear envelope reassembly, whereas ANKLE2, which does not possess a catalytic function, affects the nuclear envelope function only when depleted.

Apart from defective nuclear envelope reassembly, we also found that silencing ANKLE2 causes microtubule disorganization. Of note, T-complex protein 1 (subunit δ; TCPD), which promotes actin and tubulin folding (Dunn et al., 2001), was identified as the top ANKLE2-interacting protein in our mass spectrometry analysis. Interestingly, T-complex protein 1 subunit θ (TCPQ) was also ranked 7th in our SIRT2 interactors global score. These findings indicate that SIRT2 and ANKLE2 might cooperate in the regulation of microtubule networks at the level of actin and tubulin folding.

In addition to ANKLE2, we found interaction of SIRT2 with a series of proteins required at different levels of nuclear envelope reassembly: (1) reconstruction of nuclear envelope from ER tubules (RTN4), (2) anchoring of nuclear envelope to chromatin through inner nuclear membrane proteins [LBR and LAP1 (also known as TOR1AIP1)], and (3) RanGTPase-driven nuclear envelope fusion (RAN and RCC1). The nuclear envelope reassembly defects caused by SIRT2 depletion or overexpression might also be attributed to SIRT2 interactions with these proteins.

RTN4 is part of the reticulon family of ER-membrane shaping proteins. Reticulons generate tubular ER structures through their transmembrane domains (Hu et al., 2008; Voeltz et al., 2006; Zurek et al., 2011). During nuclear envelope reassembly from ER tubules, RTN4 needs to be gradually displaced to enable formation of flat nuclear envelope sheets (Anderson and Hetzer, 2008). Overexpression of RTN4 increases tubular ER and thereby perturbs nuclear envelope reassembly, which is contingent on membrane flattening (Anderson and Hetzer, 2008). However, the mechanism of RTN4 displacement from ER tubules during nuclear envelope reassembly is not known. Although we hypothesize that SIRT2 might regulate RTN4 displacement, we failed to notice any changes in RTN4 localization upon SIRT2 silencing in U2OS cells and could not identify any RTN4 acetylation sites by mass spectrometry (data not shown). Interestingly, RTN4 has been implicated in the nervous system physiology and pathology as a negative regulator of neuronal growth (Schwab, 2010). It remains to be tested whether the use of other cell systems such as neuronal cells will help to assign functional significance to the identified RTN4–SIRT2 interaction.

The inner nuclear membrane proteins (INM) such as the lamin B receptor (LBR) and LAP1 mediate recruitment of ER tubules onto the chromatin surface during nuclear envelope reassembly (Pyrpasopoulou et al., 1996; Ulbert et al., 2006). DNA serves as a binding site for the INM proteins that tether ER membranes to DNA during nuclear envelope reformation (Ulbert et al., 2006). Regulation of nuclear envelope reassembly by SIRT2 might therefore also involve tethering of ER membranes to chromatin through INM proteins.

As part of yet another level of nuclear envelope reassembly regulation, we validated the interaction of SIRT2 with RCC1, a guanine-nucleotide exchange factor for the small RanGTPase (Renault et al., 2001). Generation of RanGTP by RCC1 and the hydrolysis of GTP by Ran are both required for nuclear envelope reassembly (Hetzer et al., 2000). RanGTPase activity is required after the binding of the ER membranes to chromatin but before their fusion into a closed nuclear envelope (Hetzer et al., 2000). Ran acetylation at K71 has recently been shown to increase RCC1 binding but reduce RCC1-catalysed nucleotide exchange (de Boor et al., 2015). Importantly, SIRT2 specifically deacetylates K71 and thereby promotes RCC1-catalysed RanGTP formation (de Boor et al., 2015), which might be relevant in the context of nuclear envelope reassembly.

The aberrant nuclear morphology caused by SIRT2 depletion or overexpression is reminiscent of the deformed nuclei that were observed in late-passage neural stem cells or brain tissue from Parkinson's disease patients (Liu et al., 2012). Interestingly, SIRT2 has already been implicated in neurodegenerative diseases as a negative regulator of neuronal motility (Harting and Knöll, 2010; Pandithage et al., 2008). SIRT2 inhibition has been shown to have a neuroprotective effect in various Parkinson's disease models (Chen et al., 2015; Godena et al., 2014; Outeiro et al., 2007), which was linked to the axonal transport rescue through an increase in tubulin acetylation (Godena et al., 2014). Our finding that modulating the SIRT2 levels causes changes in the nuclear envelope morphology that are also observed in Parkinson's disease provides an additional insight into the mechanism of SIRT2-mediated neurodegeneration.

Deformed nuclei are also found in patients suffering from Hutchinson–Gilford progeria syndrome (HGPS) – a premature aging syndrome caused by mutations in lamin A, as well as during normal aging (Burtner and Kennedy, 2010; Ghosh and Zhou, 2014). SIRT2 was implicated in aging by regulating the mitotic checkpoint kinase BubR1 (North et al., 2014). BubR1 overexpression extends lifespan, whereas BubR1 levels decline with age and BubR1 hypomorphic mice display progeroid symptoms (Baker et al., 2004; North et al., 2014). By stabilizing BubR1 through deacetylation and protecting it from ubiquitin-mediated degradation, SIRT2 is thought to extend the lifespan of BubR1 hypomorphic mice (North et al., 2014). However, it is unclear whether SIRT2 overexpression also increases lifespan in wild-type mice (North et al., 2014) and whether SIRT2-knockout mice that do not develop tumours have a decreased lifespan (Kim et al., 2011).

The non-exhaustive list of SIRT2 interactors that we identified should instigate further exploration of the regulation of nuclear envelope and ER membrane dynamics by SIRT2, which might provide further links between SIRT2, nervous system pathologies and aging.

## MATERIALS AND METHODS

### Cell lines

Cell lines were maintained in Dulbecco's modified Eagle's Medium (DMEM; 4.5 g l^−1^ glucose) supplemented with 10% fetal bovine serum, 1% L-glutamine, 1% penicillin-streptomycin (all Sigma) under 5% CO_2_ at 37°C. HEK293T cells were used for transient transfections. To obtain mitotic fractions, HEK293T cells were treated with 330 nM nocodazole (Sigma) for 16 h. Flp-In T-Rex 293 (Life Technologies) was used for the generation of HA-STREP empty, HA-STREP SIRT2, mycBirA-SIRT2 and SIRT2-BirAmyc monoclonal stable cell lines. HA-STREP GFP Flp-In T-Rex 293 cells were a kind gift from Giulio Superti-Furga (CeMM, Vienna, Austria). HA-STREP Flp-In T-Rex 293 cells were maintained in 15 µg ml^−1^ blasticidin (InvivoGen) and 50 µg ml^−1^ hygromycin B (InvivoGen). U2OS cells stably expressing LAP2β–GFP or ANKLE2–GFP were generated by transfection of the respective plasmids and selection of stable clones in the presence of 400 µg ml^−1^ G418 (Sigma). SIRT2–LAP HeLa cells were kindly provided by the Max Planck Institute of Molecular Cell Biology and Genetics (Dresden, Germany) and maintained in 400 µg ml^−1^ G418. Transfections of HEK293T and U2OS cells were performed with polyethylenimine (PEI; Polysciences); Lipofectamine 2000 (Life Technologies) was used for the generation of the stable Flp-In T-Rex 293 cell lines.

### Plasmids and proteins

SIRT2 cDNA was obtained from Michael Potente (Angiogenesis and Metabolism Laboratory, Max Planck Institute for Heart and Lung Research, Germany) and cloned into pDONR221 (Life Technologies); HDAC1, RBBP4, MTA2 and SMRCC2 were amplified from HeLa genomic DNA and cloned into pDONR221; pDONR223 RTN4 (isoform 2), RCC1, COPB, COPG2 and RAC1 were from the human ORFeome 5.1 collection; pcDNA3.1 mycBirA was from Kyle Roux (Sanford Research, USA); LAP2β–GFP was from Jan Ellenberg (EMBL, Germany). SIRT2 was transferred into Gateway destination vector pTO_N_HA_STREP_GW_FRT (Glatter et al., 2009) for generating a stable doxycycline-inducible cell line. For *E. coli* protein expression, SIRT2 was cloned as N-terminal His_6_ fusion into pET28 (Novagen) between the NdeI and XhoI sites. For BioID, SIRT2 was cloned as an N-terminal fusion with mycBirA or a C-terminal fusion with BirAmyc_STOP_ into pDONR221 by two-step PCR. The two constructs were transferred into Gateway destination vectors pTO_N_HA_STREP_GW_FRT and pTO_C_HA_STREP_GW_FRT, respectively (Glatter et al., 2009). ANKLE2 was cloned into pCMV-EGFP-N1 between BamHI and SacI for mammalian expression and into pET102/D-TOPO by TOPO cloning for bacterial expression. siRNA-resistant pCMV ANKLE2-GFP was obtained by introducing silent mutations in three different coding regions targeted by three SMARTpool ON-TARGETplus siRNAs (5′-AAAGAACGAATAAGGGAATAC-3′; 5′-CAGAACATCGGCCGCAGTGTA-3′; 5′-CCAGCGGATCAGCTAGGA-3′). Site-directed mutagenesis was performed with the Phusion polymerase (NEB) according to the FastCloning protocol (Li et al., 2011). Catalytic cores of human CBP (amino acids 1319–1710), p300 (amino acids 1284–1673) and PCAF (amino acids 492–658) were expressed as N-terminal His_6_ fusions from the pDEST17 vector (Life Technologies). Human hMOF was expressed as an N-terminal GST fusion from pGEX-4T-1 (GE Healthcare). Proteins were expressed in *E. coli* Rosetta2 (DE3) cells (Novagen). SIRT2 and ANKLE2 were purified on HisPur Ni-NTA Resin (Pierce, Thermo Scientific) according to standard procedures. GST–hMOF was purified on glutathione–agarose (Pierce, Thermo Scientific). CBP, p300 and PCAF were purified by FPLC on a HisTrap HP column (GE Healthcare).

### Antibodies

The following antibodies were used for western blotting: rabbit anti-SIRT2 (1:1000; Sigma #S8447 or Cell Signaling #12650), rabbit anti-ANKLE2 (1:500; Atlas Antibodies #HPA003602), goat anti-RTN4 (1:1000; Nogo N18, Santa Cruz Biotechnology #sc-11027), anti-FLAG M2-peroxidase clone M2 (1:10,000; Sigma #A8592), streptavidin–HRP (1:1000; GE Healthcare #RPN1231), mouse anti-HA.11 clone 16B12 (1:1000; Covance #MMS-101R), mouse anti-Myc clone 4A6 (1:1000; Merck Millipore #05-724), rabbit anti-acetylated-lysine (1:1000; Cell Signaling #9441), mouse anti-α-tubulin clone B512 (1:5000; Sigma #T5168), mouse anti-His (1:5000; GE Healthcare #27471001), mouse anti-GFP (1:1000; Roche #11814460001). The following antibodies were used for immunofluorescence: rabbit anti-SIRT2 (1:200; Sigma #S8447), mouse anti-Myc clone 4A6 (1:500; Merck Millipore #05-724), streptavidin–Alexa-Fluor-568 (1:500; Life Technologies #S11226), rabbit anti-calnexin (1:200; a gift from Erwin Ivessa; Medical University of Vienna, Austria), mouse anti-lamin A/C (1:100; Millipore #MAB3211), mouse anti-acetylated tubulin (1:1000; Sigma #T7451). Secondary HRP-conjugated antibodies for western blotting (Jackson ImmunoResearch) were used at 1:10,000 dilution. Secondary Alexa-Fluor^®^-conjugated antibodies for immunofluorescence (Life Technologies) were used at 1:500 dilution.

### RNA interference

siRNA transfections were performed using Lipofectamine RNAiMax (Ambion, Life Technologies) according to the manufacturer's instructions. SMARTpool siRNAs for SIRT2 (ON-TARGETplus and siGENOME), ANKLE2 (ON-TARGETplus and siGENOME) and RTN4 (ON-TARGETplus) were purchased from Dharmacon. All siRNAs were used at a final concentration of 50 nM. Cells were assayed 48 h or 72 h after transfection. The level of protein knockdown was determined by western blotting using α-tubulin as a standard.

### STREP-HA purification

Five 15-cm dishes of 50% confluent HA-STREP empty, HA-STREP-GFP and HA-STREP-SIRT2 Flp-In T-Rex 293 cell lines were induced with 1 µg ml^−1^ doxycycline for 24 h prior to harvesting. Cells were harvested by scraping with cold PBS and lysed into cytoplasmic and chromatin fractions (Aygun et al., 2008). Fractions were applied to dust-free Biospin columns (BioRad) loaded with StrepTactin Sepharose (IBA) at 4°C. StrepTactin Sepharose was washed twice with the fractionation buffers containing 0.1% Triton X-100, followed by two washing steps without Triton X-100. Bound proteins were eluted with freshly prepared 2.5 mM biotin in the fractionation buffers without Triton X-100. The biotin eluate were subjected to co-immunoprecipitation with anti-HA magnetic beads (Pierce, Thermo Scientific) during a 2-h rotation at 4°C. Beads were washed six times with TBS with inhibitors. 80% of the beads were stored in 100 mM ammonium bicarbonate (ABC) and analysed by mass spectrometry following on-bead digestion, while the remaining 20% was eluted twice with glycine pH 2, neutralized with 1 M Tris-HCl pH 9.2 and used for western blotting.

### BioID

Four 15-cm dishes of Flp-In T-REx 293 cells stably carrying MycBirA–Sirt2, Sirt2–BirAMyc and empty constructs (empty vector and mycBirA) were prepared for each experimental condition. When cells reached 80% confluency, 1 µg ml^−1^ doxycycline was added for 24 h and 50 μM biotin for 16 h. For the whole-cell lysate BioID, cells were lysed in 2.4 ml lysis buffer (50 mM Tris-HCl pH 7.4, 500 mM NaCl, 0.2% SDS, 1 mM DTT and protease inhibitors); Triton X-100 was added to a final concentration of 2% and the lysates were homogenized by douncing. Samples were subsequently sonicated and centrifuged at 16,400 ***g*** for 10 min at 4°C. 200 µl of Dynabeads^®^ MyOne™ Streptavidin C1 were used per pulldown and rotated overnight at 4°C. Beads were washed twice in 1.5 ml of 2% SDS for 8 min, once in 1.5 ml wash buffer 2 (0.1% deoxycholic acid, 1% Triton X-100, 1 mM EDTA, 500 mM NaCl and 50 mM Hepes pH 7.5), once in 1.5 ml wash buffer 3 (0.5% deoxycholic acid, 0.5% NP-40, 1 mM EDTA, 250 mM LiCl and 10 mM Tris-HCl pH 7.4) and once in 1.5 ml 50 mM Tris-HCl pH 7.4. 10% of the beads were saved for further analysis by western blotting. The samples were centrifuged at 6000 ***g*** for 5 min, then the beads were resuspended in 100 µl of 100 mM ammonium bicarbonate, frozen in liquid nitrogen and stored at −80°C until processing for mass spectrometry analysis. The beads saved for western blot analysis were resuspended in 40 µl of sample buffer.

### GFP and HA co-immunoprecipitation

For anti-GFP and anti-HA co-immunoprecipitation, cells were harvested by scraping and cell pellets were lysed in the lysis buffer containing 50 mM Tris-HCl pH 8, 150 mM NaCl, 1% Triton X-100, 1 mM DTT, 50 U/ml benzonase (Novagen), protease inhibitors (Complete Mini Protease Inhibitor Cocktail Tablets, EDTA-free; Roche), 5 µM trichostatin A (TSA; Sigma), 20 mM nicotinamide (NAM; Sigma) for 1 h with rotation at 4°C. 10 mM NEM (Sigma), 2 mM Na_3_VO_4_ (Sigma) and 1 mM PMSF (Sigma) were added for PTM analysis. Cell lysates were rotated with anti-GFP antibodies coupled to magnetic beads (GFP-Trap_M, Chromotek) for 2 h at 4°C. The beads were washed five times with the lysis buffer without Triton. Anti-HA magnetic beads (Pierce) were equilibrated by washing the beads twice with TBS. Lysates were incubated with the beads for 2 h at 4°C with rotation. The beads were washed three times with the lysis buffer and eluted with glycine pH 2.

### Mass spectrometry

Nano liquid chromatography mass spectrometry (LC-MS) analysis was performed with the UltiMate 3000 HPLC RSLC nano system (Thermo Scientific) coupled to a Q Exactive mass spectrometer (Thermo Scientific), equipped with a Proxeon nanospray source (Thermo Scientific). Peptides were loaded onto a trap column (PepMap C18, 5 mm×300 μm ID, 5 μm particles, 100 Å pore size; Thermo Scientific) followed by the analytical column (PepMap C18, 500 mm×75 μm ID, 3 μm, 100 Å; Thermo Scientific). The elution gradient started with the mobile phases: 98% A (water:formic acid, 99.9:0.1, v/v) and 2% B (water:acetonitrile:formic acid, 19.92:80:0.08, v/v/v), increased to 35% B over the next 120 min followed by a 5-min gradient to 90% B, stayed there for 5 min and decreased in 5 min back to the gradient 98% A and 2% B for equilibration at 30°C. The Q Exactive mass spectrometer was operated in data-dependent mode, using a full scan followed by MS/MS scans of the 12 most abundant ions. For peptide identification, the RAW-files were loaded into Proteome Discoverer (version 1.4.0.288, Thermo Scientific). The resultant MS/MS spectra were searched using Mascot 2.2.07 (Matrix Science) against the Swissprot protein sequence database, using the taxonomy human. β-methylthiolation on cysteine was set as a fixed modification, oxidation on methionine, acetylation on lysine, phosphorylation on serine, threonine and tyrosine, mono- and dimethylation on lysine and arginine, trimethylation on lysine and ubiquitinylation on lysine were set as variable modifications. The peptide mass tolerance was set to ±5 ppm and the fragment mass tolerance to ±0.03 Da. The maximal number of missed cleavages was set to 2. The result was filtered to a 1% false-discovery rate (FDR) using Percolator algorithm integrated in Proteome Discoverer (Elias and Gygi, 2007). The localization of the sites of variable modifications within the peptides was performed with the tool ptmRS, integrated in Proteome Discoverer and based on phosphoRS (Taus et al., 2011).

### Analysis of mass spectrometry data

SAINT-MS1 was used as a statistical tool to determine the probability of protein–protein interactions (Choi et al., 2012a). Prior to analysis with SAINT-MS1 (Choi et al., 2012a) the label-free quantification data was cleaned by removing bait and common laboratory contaminants (Choi et al., 2012b). The controls (empty vector and GFP for TAP or empty vector and mycBirA for BioID) were used simultaneously to estimate the parameters of the false interaction probability distributions. SAINT-MS1 was run for each method and fraction separately with 5000 and 10,000 burn-in and sampling iterations, respectively. Protein areas were normalized to obtain a median protein ratio of one between samples. Fold changes were calculated based on these normalized protein areas. The global score was calculated as the sum of measured interactions stringently above the SAINT-MS1 probability and a fold change of 0.98 and 10, respectively. Each method that satisfied the above criteria contributed one point to the global score. Additionally, for each method contributing one point, the average method probability was calculated. Method probabilities refer to SAINT probabilities >0.98 for each of the three possible controls (empty vector, GFP or empty vector+GFP; empty vector, mycBirA or empty vector+mycBirA). Probabilities <0.98 were omitted. The product of these probabilities for all contributing methods was added to the global score, resulting in enhanced granularity. In brief, the global score is the sum of the number of methods and indicates an interaction and a granularity constant calculated on the basis of the SAINT-MS1 probability.

Analysis of PTMs was performed in Microsoft Excel 2013. Only peptide spectrum matches (PSM) with a false discovery rate (FDR) of 1% were used in the analysis. The PSMs were grouped by sequence and modifications. For each modified PSM group [e.g. ANSYK(ac)NPR], the unmodified counterpart [e.g. ANSYKNPR] was searched and then the ratio calculated as shown in the formula:
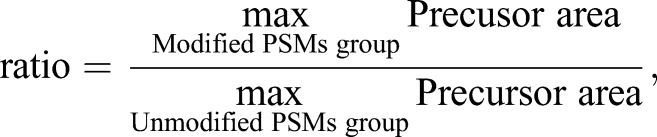
where ‘0’ indicates that only the modified peptide could be identified or that no modification for the given residue was found, which precludes abundance determination. The position of the modification of each modified group was matched onto the protein sequence. The average abundance ratio is the mean ratio between two or four replicates. Samples were compared by calculating the log_2_ ratio. Standard deviations were calculated to estimate variability.

### Immunofluorescence

Cells were grown on sterile glass coverslips or glass bottom dishes (Greiner CELLview™), rinsed with PBS, fixed in 4% paraformaldehyde for 10 min and permeabilized using 0.5% Triton X-100 in PBS for 5 min. Cells were subsequently blocked in 0.5% gelatin or 3% BSA+0.1% Triton in PBS for 15 min, incubated with primary antibodies for 45 min or at 4°C overnight, and washed and probed with the appropriate secondary antibodies conjugated to Alexa Fluor 488 and/or Alexa Fluor 568 (Molecular Probes) for 45 min. Cells were stained with DAPI and mounted in 60% glycerol in 20 mM Tris-HCl pH 8. Images were taken with a confocal laser-scanning microscope LSM700 (Zeiss). Digital images were analysed and adjusted for brightness and contrast using the software LSM-Image-Browser (Zeiss).

### *In vitro* acetylation and deacetylation assays

The *in vitro* acetylation assay was performed by incubating 0.05, 0.5 or 5 µg human His_6_-tagged ANKLE2 with 0.5 µg of CBP, p300, PCAF or hMOF acetyltransferase in 50 µl of 10 µM acetyl-CoA (Sigma), 50 mM Tris-HCl pH 8, 10% glycerol, 0.1 mM EDTA, 1 mM DTT and 1 mM PMSF for 2 h at 30°C with rotation. The reaction was stopped by the addition of 20 µl SDS sample buffer and 20 µl was loaded on the gel. The *in vitro* deacetylation assay was performed by stopping the above acetylation reaction with 20 µM anacardic acid and adding 0.5 or 5 µg His_6_-tagged wild-type SIRT2 or its catalytically inactive H150Y mutant in the presence of 200 μM NAD and incubating for 90 min at 37°C.

### Statistics

Error bars represent the s.e.m. estimated from two to four independent experiments. Statistical significance was calculated using a two-tailed Student's *t*-test. *P*-values smaller than 5% were considered statistically significant and indicated with asterisks (*P<0.05; **P<0.01; ***P<0.001).
